# Interactive Training of the Emergency Medical Services Improved Prehospital Stroke Recognition and Transport Time

**DOI:** 10.3389/fneur.2022.765165

**Published:** 2022-04-07

**Authors:** Lukas Sveikata, Kazimieras Melaika, Adam Wiśniewski, Aleksandras Vilionskis, Kȩstutis Petrikonis, Edgaras Stankevičius, Kristaps Jurjans, Aleksandra Ekkert, Dalius Jatužis, Rytis Masiliūnas

**Affiliations:** ^1^J. Philip Kistler Stroke Research Center, Department of Neurology, Massachusetts General Hospital, Harvard Medical School, Boston, MA, United States; ^2^Institute of Cardiology, Medical Academy, Lithuanian University of Health Sciences, Kaunas, Lithuania; ^3^Faculty of Medicine, Vilnius University, Vilnius, Lithuania; ^4^Department of Neurology, Collegium Medicum in Bydgoszcz, Nicolaus Copernicus University in Toruń, Bydgoszcz, Poland; ^5^Clinic of Neurology and Neurosurgery, Institute of Clinical Medicine, Vilnius University, Vilnius, Lithuania; ^6^Stroke Center, Republican Vilnius University Hospital, Vilnius, Lithuania; ^7^Department of Neurology, Lithuanian University of Health Sciences, Kaunas, Lithuania; ^8^Department of Neurology and Neurosurgery, Riga Stradins University, Riga, Latvia; ^9^Department of Neurology, Pauls Stradins Clinical University Hospital, Riga, Latvia; ^10^Center of Neurology, Vilnius University, Vilnius, Lithuania

**Keywords:** training, triage, emergency medical services (EMS), transient ischemic attack (TIA), prehospital/EMS, stroke

## Abstract

**Background and Purpose:**

Acute stroke treatment outcomes are predicated on reperfusion timeliness which can be improved by better prehospital stroke identification. We aimed to assess the effect of interactive emergency medical services (EMS) training on stroke recognition and prehospital care performance in a very high-risk cardiovascular risk population in Lithuania.

**Methods:**

We conducted a single-center interrupted time-series study between March 1, 2019 and March 15, 2020. Two-hour small-group interactive stroke training sessions were organized for 166 paramedics serving our stroke network. We evaluated positive predictive value (PPV) and sensitivity for stroke including transient ischemic attack identification, onset-to-door time, and hospital-based outcomes during 6-months prior and 3.5 months after the training. The study outcomes were compared between EMS providers in urban and suburban areas.

**Results:**

In total, 677 suspected stroke cases and 239 stroke chameleons (median age 75 years, 54.8% women) were transported by EMS. After the training, we observed improved PPV for stroke recognition (79.8% *vs*. 71.8%, *p* = 0.017) and a trend of decreased in-hospital mortality (7.8% *vs*. 12.3, *p* = 0.070). Multivariable logistic regression models adjusted for age, gender, EMS location, and stroke subtype showed an association between EMS stroke training and improved odds of stroke identification (adjusted odds ratio [aOR] 1.6 [1.1–2.3]) and onset-to-door ≤ 90 min (aOR 1.6 [1.1–2.5]). The improvement of PPV was observed in urban EMS (84.9% *vs*. 71.2%, *p* = 0.003), but not in the suburban group (75.0% *vs*. 72.6%, *p* = 0.621).

**Conclusions:**

The interactive EMS training was associated with a robust improvement of stroke recognition, onset to hospital transport time, and a trend of decreased in-hospital mortality. Adapted training strategies may be needed for EMS providers in suburban areas. Future studies should evaluate the long-term effects of the EMS training and identify optimal retraining intervals.

## Introduction

Stroke is a life-threatening condition in which prompt and accurate diagnosis is essential for successfully implementing reperfusion therapies (1). Emergency medical services (EMS) play a crucial role in early recognition of stroke, as they are the first-line providers in about two-thirds of cases (2). Although EMS use by stroke patients is associated with earlier emergency department (ED) arrival, quicker evaluation, and more rapid treatment, how healthcare providers respond to stroke remains an essential factor in explaining prehospital delays (1). The process of clinically identifying a stroke is still the most significant challenge for EMS, as a percentage of stroke mimics reaches up to 50% (3, 4). Consequently, stroke mimics utilize the limited resources of acute stroke care pathways that might otherwise be directed toward the actual stroke patients who may benefit from acute time-sensitive revascularization therapies the most (5). Of concern, stroke mimic number in stroke care systems is expected to rise due to demographic changes in the coming decades (6). Therefore, it is crucial to improve the EMS performance in early stroke recognition. Fast and correct stroke diagnosis facilitates an early transfer to stroke-ready hospitals, reduces the volume of stroke mimics, and improves outcomes of acute stroke.

Intensive efforts are made to improve the quality of early stroke care. Training programs for EMS staff in simulated neurological environments increase knowledge on stroke recognition and awareness of time-sensitive medical emergencies (1, 7–12). The hospital prenotification has improved in-hospital timeliness metrics and increased intravenous thrombolysis (IVT) rates (13). In addition, prehospital stroke scales and screening methods for EMS staff have been introduced to allow for a more objective stroke identification (e.g., Face Arm Speech Time test, Los Angeles Motor Scale, Cincinnati Prehospital Stroke Scale) (14, 15). Moreover, specific scales for large vessel occlusion stroke were developed to facilitate the identification of candidates for endovascular therapy (EVT) (16). Given the changing landscape of prehospital stroke identification, a continuous educational effort is required to ensure optimal implementation of prehospital stroke protocols.

Stroke education interventions in prehospital care provided mixed results. A large multicenter randomized control trial in the United Kingdom did not show any benefit on the IVT rate. Surprisingly, the onsite care duration was prolonged in the EMS group that applied an enhanced stroke assessment protocol (17). On the other hand, several interventions increased the accuracy of stroke identification, the number of patients who underwent reperfusion therapy, and significantly reduced the time from the symptom onset to hospital arrival (7–9, 11, 18). Furthermore, the duration of the training effects remains unknown (19). Finally, the paucity of studies in very high cardiovascular risk populations, such as the Baltic countries, urged us to investigate prehospital stroke care intervention in Lithuania (20). Following the European (21) and North American guidelines (13), it is crucial to systematically assess the effectiveness of specific stroke education interventions and maintain the continuity of EMS education.

This study aimed to prospectively evaluate the effect of interactive EMS training on stroke recognition accuracy and the continuum of stroke care metrics. Second, we hypothesize that the EMS training effect might differ in the communities served and compare the training effect in urban and suburban locations.

## Methods

### Study Design

We used an interrupted time-series design (22) to examine the impact of interactive EMS stroke training on EMS and hospital-based performance measures. We evaluated the positive predictive value (PPV) and sensitivity for identifying stroke patients, onset-to-door (OTD) ≤ 90 min rate, and hospital-based outcomes, including door-to-CT ≤ 30 min rate, reperfusion therapy, door-to-needle ≤ 30 min rate, and in-hospital mortality. We compared these variables between two periods−6 months before and 3.5 months after the interactive EMS training. The EMS personnel were blinded to the assessment.

The study was approved by the Vilnius Regional Biomedical Research Ethics Committee and conducted following the Declaration of Helsinki. The manuscript complies with STROBE guidelines for observational research.

### Setting

This single-center study was conducted in Vilnius University Hospital (VUH) from 1 March 2019 to 16 March 2020, terminated earlier due to an unanticipated state-wide COVID-19 lockdown (23). VUH is one of the two comprehensive stroke centers (CSC) in Eastern Lithuania with a catchment population of 945,000^1^, served by one EMS agency in urban and seven in suburban municipalities (24). The EMS response team consisted of a two-person team—paramedic and driver-paramedic. The EMS agencies were staffed by 331 specialists (217 in urban and 114 in suburban locations) and transported ≈20,400 patients.^2^ The paramedics had prior training in nursing (307, 92.7%) or medicine (24, 7.3%).

The post-training period coincided with the change in national stroke triage guidelines, implemented on January 1, 2020. The new regulations affected the workflow of suburban EMS as it required direct transport of all suspected stroke cases to the stroke-ready hospitals with IVT or EVT capability, bypassing regional hospitals irrespective of the time from symptom onset.^3^

### Study Population

**Figure 1:**
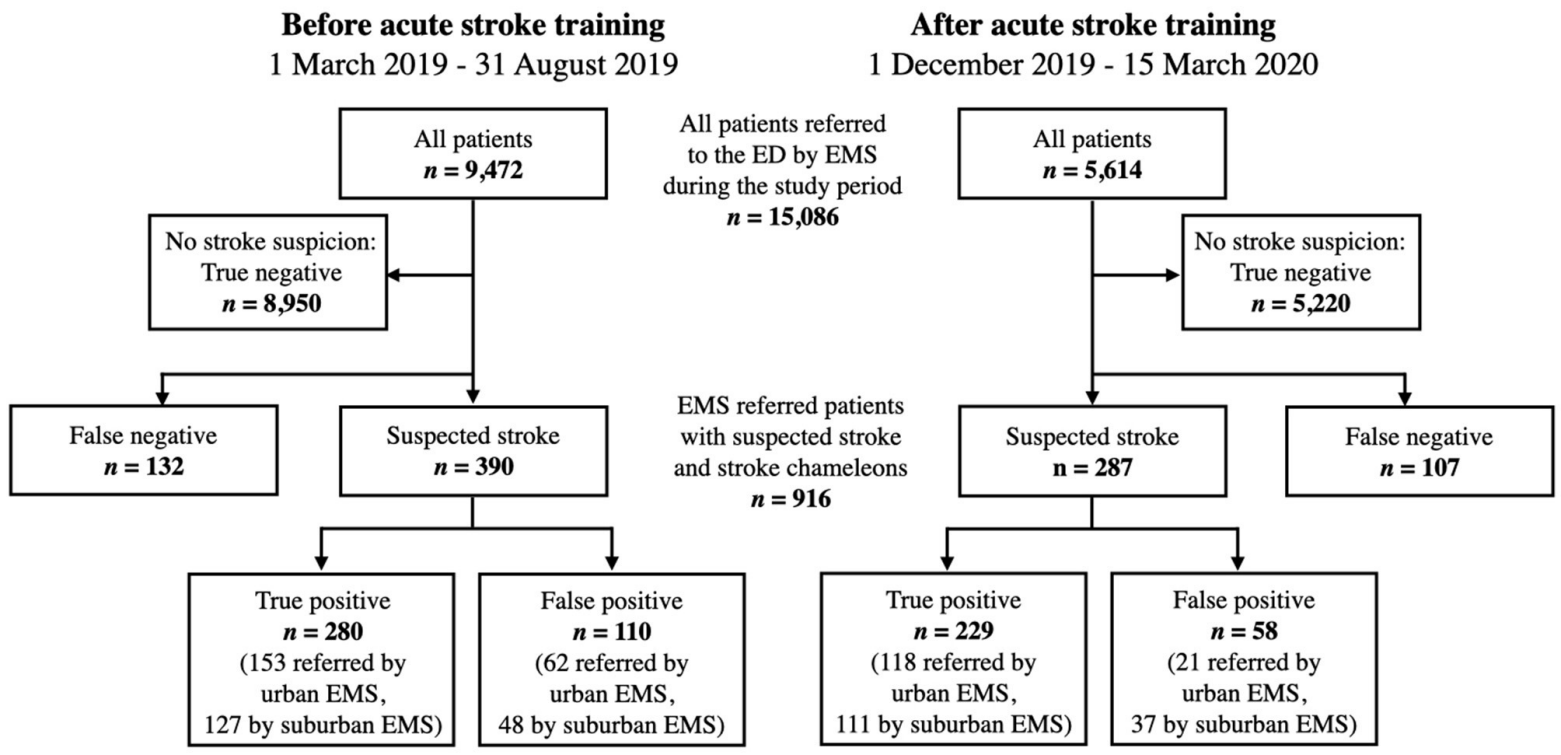
Flowchart of the study population. Patients were referred to the emergency department (ED) of Vilnius University Hospital by emergency medical services (EMS) between March 1, 2019 and March 15, 2020.

We collected data of suspected stroke or transient ischemic attack (TIA) patients referred by the EMS to the VUH ED. Secondary transfers from other hospitals and in-hospital strokes were not included. We also collected data on false negatives, that is, stroke cases that were not identified by the EMS. EMS used the Face Arm Speech Time test (FAST) for the identification of suspected strokes (25). Overall, 15,086 patients were referred to the ED by the EMS, of whom 916 patients with EMS suspected or hospital confirmed strokes were included in the analysis (Figure 1). Stroke case ascertainment was done after arrival at the hospital by an attending neurologist after a complete stroke work-up. We did not include cases admitted during the 3-month training period.

### Interactive EMS Training

Twelve 2-h interactive prehospital stroke recognition training sessions were held in the Neurology Department of VUH over 3 months (from September to November 2019). Interactive training sessions were given by stroke neurologists from the Lithuanian Stroke Association. Each training session was limited to 20 EMS staff members. In total, 166 out of 331 (50.2%) paramedics working in our stroke network participated in the training. The training curriculum was based on the publicly available ANGELS initiative's e-learning course for stroke education for emergency medical teams^4^ adapted for local needs and in-person delivery (available online^5^). The EMS stroke training covered stroke epidemiology, pathophysiology, acute stroke treatment, and outcomes, emphasizing the time-sensitive aspects of acute stroke care. The EMS staff was trained to recognize stroke with the FAST test and identify the major stroke syndromes and stroke mimics. Additionally, participants received an update on prehospital acute stroke management. The presentation emphasized the importance of last known well (LKW) documentation, glucose check, minimizing the on-scene time, and hospital prenotification, followed by an interactive discussion.

### Data Collection

Demographic and clinical characteristics such as age, gender, stroke type, daily stroke volume, type of reperfusion therapy, acute stroke care timeliness metrics (onset-to-door, door-to-needle, door-to-groin), and the National Institutes of Health Stroke Scale (NIHSS) scores at admission and discharge were collected for all confirmed strokes and stroke alerts referred by EMS. True positives were defined as EMS-suspected strokes followed by in-hospital confirmation of stroke (ischemic stroke, intracerebral hemorrhage, or subarachnoid hemorrhage) or TIA after a complete neurologic evaluation, including neuroimaging by CT or MRI. False positives, or stroke mimics, were defined as stroke alerts given an alternative diagnosis after a full assessment. Furthermore, we collected information on false negative cases, termed stroke chameleons. The NIHSS score was documented only for patients who were considered for reperfusion therapy.

### Statistical Analysis

We compared categorical variables using the χ^2^ test and Fisher's exact test, as appropriate. Based on their Gaussian distribution, the quantitative variables were compared using the Student's *t*-test or Mann–Whitney *U* test. Baseline characteristics and outcome measures were compared before and after the training and based on EMS location strata (urban *vs*. suburban). The 95% confidence intervals (CI) were calculated, where applicable.

Before the training, baseline trends in monthly EMS performance and hospital-based outcomes were assessed using univariate linear regression and the χ^2^ test for trend. We performed multivariable logistic regression models to assess the association between the training and EMS performance and in-hospital outcome measures. To account for potential confounding effects of age, gender, EMS location, and stroke subtype where appropriate, we used the hybrid backward/forward stepwise selection using the Akaike information criterion (26), removing variables with a nonsignificant (*p* > 0.05) association. Age was forced into all models as an *a priori* confounder. *P* < 0.05 (two-sided) was considered statistically significant. IBM SPSS Statistics 23.0 software (IBM Corp., Armonk, NY, United States) and R version 3.6.2 were used for statistical analyses.

## Results

We enrolled 916 patients with a median age of 75 (interquartile range: 66–82) years, of which 502 (54.8%) were female. In total, 677 suspected strokes (73.9%) were admitted to the ED, comprising 509 true positives (55.6%) and 168 false positives (18.3%). In contrast, EMS did not recognize 239 (26.1%) strokes, labeled false negatives or stroke chameleons. The study groups before and after the training were balanced in terms of demographics, stroke subtype, and baseline NIHSS (Table 1). Urban EMS providers transported 500 patients (54.6%), whereas suburban EMS transported 416 (45.4%).

**Table 1:** Baseline characteristics and outcomes of emergency medical services suspected stroke admissions.

	**All patients (*n* = 916)**	**Admitted before training (*n* = 522)**	**Admitted after training (*n* = 394)**	***P*-value**	**Referred by urban EMS (*n* = 500)**	**Referred by suburban EMS (*n* = 416)**	***P-*value**
Median age, years (IQR)	75 (66–82)	74 (65–82)	75 (66–82)	0.596	75 (66–82)	75 (65–82)	0.340
Female sex, *n* (%)	502 (54.8)	276 (52.9)	226 (57.4)	0.177	272 (54.4)	230 (55.3)	0.788
Confirmed strokes, *n* (%)	748 (81.7)	412 (78.9)	336 (85.3)		417 (83.4)	331 (79.6)	
Ischemic stroke	606 (66.2)	339 (64.9)	267 (67.8)	0.371	335 (67.0)	271 (65.1)	0.555
Hemorrhagic stroke	86 (9.4)	46 (8.8)	40 (10.2)	0.491	48 (9.6)	38 (9.1)	0.810
ICH	68 (7.4)	37 (7.1)	31 (7.9)	0.656	38 (7.6)	30 (7.2)	0.823
SAH	18 (2.0)	9 (1.7)	9 (2.3)	0.545	10 (2.0)	8 (1.9)	0.933
Transient ischemic attack	56 (6.1)	27 (5.2)	29 (7.4)	0.171	34 (6.8)	22 (5.3)	0.342
Stroke mimics, *n* (%)	168 (24.8)	110 (28.2)	58 (20.2)	0.017	83 (23.4)	85 (26.3)	0.388
Stroke chameleons, *n* (%)	239 (32.0)	132 (32.0)	107 (31.8)	0.955	146 (35.0)	93 (28.1)	0.044
**Daily volume, median (IQR)**							
Stroke alerts	2 (1-3)	2 (1-3)	3 (2-4)	0.002	1 (0-2)	1 (0-2)	0.275
Confirmed strokes	2 (1-4)	2 (1-3)	3 (2-4)	<0.001	1 (1-2)	1 (1-2)	0.005
Reperfusion of ischemic strokes, *n* (%)	203 (33.5)	126 (37.2)	77 (28.8)		110 (32.8)	93 (34.3)	
Not eligible	403 (66.5)	213 (62.8)	190 (71.2)	0.031	225 (67.2)	178 (65.7)	0.701
IVT	97 (16.0)	54 (15.9)	43 (16.1)	0.953	59 (17.6)	38 (14.0)	0.231
EVT	86 (14.2)	62 (18.3)	24 (9.0)	0.001	41 (12.2)	45 (16.6)	0.126
Combined treatment	20 (3.3)	10 (2.9)	10 (3.7)	0.586	10 (3.0)	10 (3.7)	0.629
**Median timeliness metrics, min (IQR)**							
Onset-to-door^†^	114 (75-198)	119 (78-205)	110 (74-196)	0.606	93 (67-159)	137 (89-269)	<0.001
Door-to-needle	41 (29-59)	41.5 (31-58)	41 (29-60)	0.850	46 (31-63)	37.5 (28-51)	0.078
Door-to-groin	81.5 (61-102)	73.5 (61-100)	90 (65-110)	0.206	88 (60-107)	78 (61-98)	0.517
Baseline NIHSS, median (IQR)^‡^	8 (4–15)	9 (5–15)	7 (4–15)	0.072	8 (4–15)	8 (5–15)	0.643
Discharge NIHSS, median (IQR)^‡^	3 (1–5)	3 (1–5)	3 (1–5)	0.962	3 (1–4)	3 (1–5)	0.360

*IQR, interquartile range; ICH, intracerebral hemorrhage; SAH, subarachnoid hemorrhage; IVT, intravenous thrombolysis; EVT, endovascular treatment; NIHSS, National Institutes of Health Stroke Scale.*

*^†^Only patients with established onset of symptoms are included (n = 433).*

*^‡^Baseline (n = 343) and discharge NIHSS (n = 172) are reported only for ischemic stroke patients who were considered for reperfusion therapy*.

### Demographic and Clinical Characteristics Before and After the Training

More daily stroke alerts (3 [2–4] *vs*. 2 [1–3], *p* = 0.002) and confirmed strokes (3 [2–4] *vs*. 2 [1–3], *p* <0.001) were observed in the post-training period. However, proportionally fewer patients were eligible for reperfusion therapy (28.8% post-training *vs*. 37.2% pre-training, *p* = 0.031) due to a decreased rate of endovascular therapy compared to the pre-training period (9.0% *vs*. 18.3%, *p* = 0.001). No significant differences in IVT and combined treatment groups were observed.

The median onset-to-door time (110 [74–196] min *vs*. 119 [78–205] min, *p* = 0.606) improved numerically after the training but did not reach statistical significance. The door-to-needle, door-to-groin times, and discharge NIHSS did not differ significantly before and after the stroke training.

We did not identify any trends in EMS performance or prehospital care metrics during the 6 months before the EMS training (Table 2); thus, next, we assessed the impact of EMS training on these metrics.

**Table 2:** Trends in emergency medical services performance and hospital-based outcomes during the 6 months before the training.

**Performance**	**Regression**	***P*-value^‡^**
	**coefficient^†^**	
EMS recognized stroke patients	−0.0173	0.527
(sensitivity)		
PPV for identification of	−0.0027	0.692
stroke patients		
Onset-to-door ≤ 90 min	−0.0131	0.924
Door-to-CT ≤ 30 min	−0.0035	0.848
IVT rate	−0.0054	0.425
Door-to-needle time ≤ 30 min	−0.0169	0.415
In-hospital mortality	0.0028	0.606

*PPV, positive predictive value; CT, computed tomography; IVT, intravenous thrombolysis.*

*^†^Linear regression coefficient for the proportion of cases with each outcome during 1-month intervals.*

*^‡^^χ^2 or Fisher's Exact Test for trend, as appropriate*.

### EMS Training Effect

In the pairwise comparison, the PPV for the identification of acute stroke patients was significantly higher in the post-training period (79.8% [75.1–84.4] *vs*. 71.8% [67.3–76.3], *p* = 0.017). Notably, however, the proportion of false negatives and the EMS recognized stroke patients (sensitivity) did not differ before and after the intervention (Table 3). Although there was a weak trend for improvement in door-to-needle times and in-hospital mortality, there was no statistically significant difference in other hospital-based outcomes before and after the EMS training.

**Table 3:** Emergency medical services performance and hospital-based outcomes among 916 suspected or confirmed strokes before and after the training.

**Performance**	**All patients**	**Before training**	**After training**	***P*-value**
	**(95% CI)**	**(95% CI)**	**(95% CI)**	
EMS recognized stroke patients (sensitivity)	68.0% (64.6–71.3)	68.0% (63.3–72.3)	68.2% (63.0–72.9)	0.955
PPV for identification of stroke patients	75.2% (71.9–78.4)	71.8% (67.3–76.3)	79.8% (75.1–84.4)	0.017
Onset-to-door ≤ 90 min	37.2% (32.8–41.8)	33.7% (28.2–39.8)	42.0% (35.0–49.3)	0.079
Door-to-CT ≤ 30 min^†^	84.2% (78.6–88.6)	84.1% (76.8–89.5)	84.4% (74.7–90.9)	0.956
IVT rate^‡^	19.3% (16.4–22.6)	18.9% (15.1–23.4)	19.9% (15.5–25.1)	0.764
Door-to-needle time ≤ 30 min	27.4% (20.1–36.1)	23.4% (14.8–35.1)	32.1% (21.1–45.5)	0.297
In-hospital mortality^§^	10.5% (8.4–13.2)	12.3% (9.4–16.0)	7.8% (5.1–11.8)	0.070

*CI, confidence interval; PPV, positive predictive value; CT, computed tomography; IVT, intravenous thrombolysis.*

*^†^Included stroke patients, who underwent reperfusion treatment (n = 203).*

*^‡^Only ischemic stroke patients (n = 606).*

*^§^Only hospitalized stroke patients (n = 636)*.

Multivariable logistic regression showed improved odds of stroke identification (PPV) (Table 4), which remained significant after adjusting for age, gender, and EMS location (adjusted odds ratio (aOR) 1.6 [1.1–2.4]). Furthermore, we observed improved odds of patient arrival within 90 min of stroke onset (aOR 1.6 [1.1–2.5]), driven by an improvement in OTD ≤ 90 min time in urban EMS (56.8% [46.4–66.7] post-training *vs*. 41.1% [33.5–49.0] pre-, *p* = 0.019).

**Table 4:** Logistic regression models showing the association between emergency medical services training and acute stroke care performance measure and hospital-based outcomes.

**Outcome**	**Unadjusted**	**Adjusted^†^**
	**OR (95% CI)**	**OR (95% CI)**
EMS recognized stroke patients	1.0 (0.7–1.4)	1.0 (0.7–1.4)^‡^
(sensitivity)		
PPV for identification of	1.6 (1.1–2.2) *	1.6 (1.1–2.4) *
stroke patients		
Onset-to-door time ≤ 90 min	1.4 (1.0–2.1)	1.6 (1.1–2.5)^‡^*
Door-to-CT time ≤ 30 min	1.0 (0.4–2.9)	0.8 (0.3–2.4)
IVT rate	1.1 (0.7–1.6)	1.1 (0.7–1.6)
Door-to-needle time ≤ 30 min	1.5 (0.7–3.5)	1.5 (0.6–3.5)
In-hospital mortality	0.6 (0.3–1.0)	0.6 (0.4–1.1)^‡^

*OR, odds ratio; CI, confidence interval; PPV, positive predictive value; CT, computed tomography; IVT, intravenous thrombolysis.*

*^†^Adjusted for age, gender, and EMS location (urban vs. suburban).*

*^‡^Adjusted for age, gender, EMS location, and stroke type.*

*P <0.05.

### Urban vs. Suburban EMS

EMS-referred patients from urban and suburban areas did not differ in demographic characteristics, acute stroke types, baseline and discharge stroke severity, and eligibility for reperfusion therapy (Table 1). Although there were more overall daily confirmed strokes referred by urban EMS (1 [1–2] *vs*. 1 [0–2], *p* = 0.005), there was no difference in the proportion of suspected strokes *vs*. total patients transported by urban and suburban EMS; thus, indicating similar suspected stroke prevalence in both groups.

**Figure 2:**
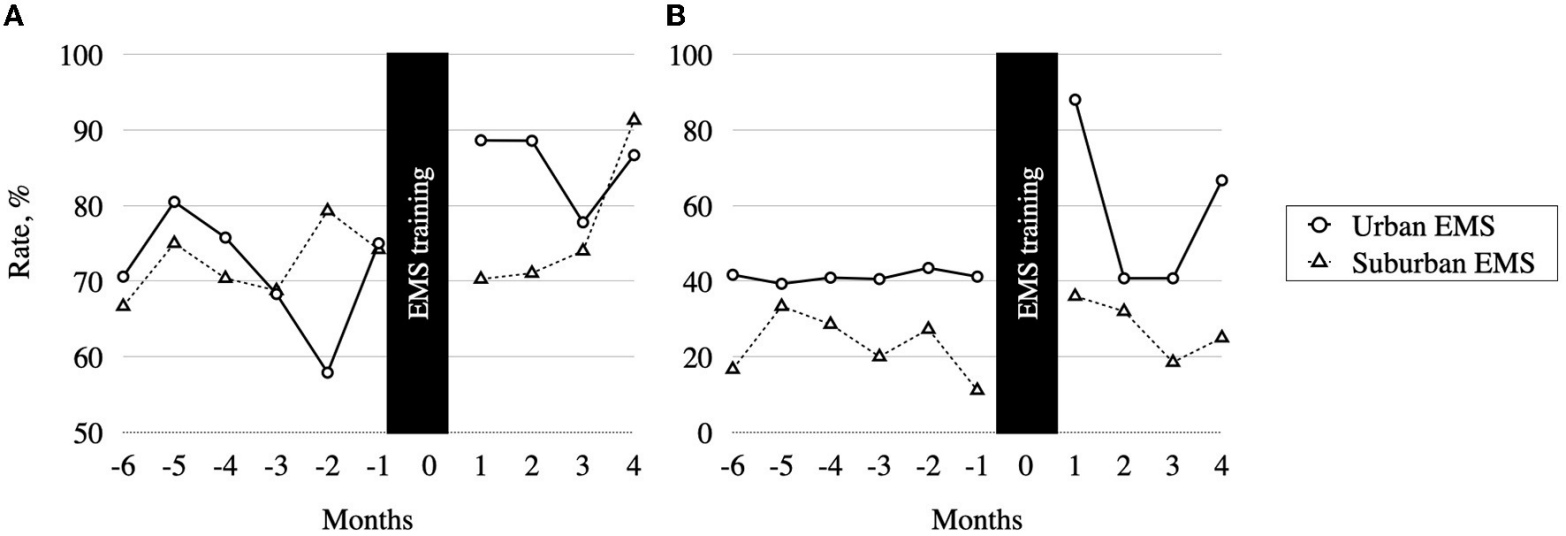
Emergency medical services (EMS) performance before and after the EMS training. **(A)** Positive predictive value (PPV) for identification of stroke patients. **(B)** Onset-to-door time ≤ 90 min rate stratified by EMS location.

There was no significant baseline difference in PPV values between urban and suburban EMS (Figure 2). However, after the training, the PPV improved in the urban EMS group (84.9% [78.9–90.8] *vs*. 71.2% [65.1–77.2], *p* = 0.003), but not in the suburban EMS (75.0% [68.0–82.0] *vs*. 72.6% [66.0–79.2], *p* = 0.621). Marginally more stroke chameleons were referred by urban than suburban EMS (35.0% [30.6–39.7] *vs*. 28.1% [23.5–33.2], *p* = 0.044), indicating lower sensitivity in the urban EMS group. However, there was no significant difference in sensitivity before and after the training within each EMS group.

Shorter overall median onset-to-door time was observed in patients referred by urban EMS (93 [67–159] min *vs*. 137 [89–269] min, *p* < 0.001), and more urban patients reached the CSC within 90 min (46.9% [40.6–53.2] 25.3% [19.7–31.8], *p* < 0.001) compared to the suburban EMS. After the training, there was a weak trend for improvement of the absolute onset-to-door time (84.5 min *vs*. 108.0 min, *p* = 0.074) and a significant improvement in onset-to-door ≤ 90 min rate in the urban EMS (70.5% [60.2–79.0] *vs*. 41.1% [33.5–49.0], *p* = 0.019), but not in the suburban EMS group (28.0% [19.9–37.8] *vs*. 22.8% [15.7–31.9], *p* = 0.406) (Figure 2).

## Discussion

We have several main findings from this prospective interrupted time-series study evaluating the effect of interactive EMS training on prehospital stroke care. First, we found a sustained improvement of prehospital stroke recognition during at least four consecutive months after the training. Second, we found an improved rate of timely transfers of suspected strokes to the hospital, demonstrating the overall benefit of EMS training on the continuum of prehospital care. Third, we found a trend of decreased in-hospital mortality that could be related to more timely stroke patient transport to the hospital. Finally, the training effect was more pronounced in the urban EMS group and, thus, we discuss the possible reasons and implications.

We found fewer stroke mimics in the post-training period without an increase in the false negative rate. Thus, increasing the PPV did not result in suboptimal triage of strokes, nor did it deprive stroke patients of time-sensitive revascularization treatment. The improvement of PPV was driven by a reduced rate of stroke mimics in the urban EMS group. One of the reasons for significantly improved PPV in the urban but not the suburban EMS group could be the implementation of new national regulation of prehospital stroke triage on January 1, 2020, that partially overlapped with the post-training period. According to the new law, suspected stroke patients were transferred directly to stroke-ready hubs bypassing primary evaluation in the regional hospitals irrespective of their LKW time. The new guidelines were designed to improve access to reperfusion therapy for stroke patients in the suburban regions. However, the stroke triage pathway change may have increased the false positive rate in the suburban EMS group as they transported more suspected stroke cases directly to the CSC instead of the regional hospitals. Thus, we speculate that the weak trend of PPV improvement in the suburban EMS group reflects the effect of EMS training offsetting the expected dip of PPV in the suburban EMS group. Another possible explanation could be differences in stroke knowledge between urban and suburban paramedics before the training or other variables, such as differing socioeconomic status, comorbidities, or secular trends, not evaluated in this study.

The increase in PPV after the training is clinically relevant because it can help reduce the false positive cases overflowing the acute stroke care pathways. Optimal utilization of the frontline stroke care and neuroimaging resources is particularly relevant during peak hours of stroke incidence, such as the morning hours (27) or public health emergencies, as was the case during the COVID-19 pandemic (23). Therefore, continuous efforts are crucial to ensure optimal prehospital stroke identification.

Previous studies have shown that a brief educational EMS intervention could substantially improve EMS knowledge of prehospital stroke scales, prenotification compliance, and field triage protocols (10, 28). Moreover, a recent prospective study by Oostema et al. assessed the real-world impact of EMS training on prehospital stroke recognition and found that an online EMS education module coupled with performance feedback was associated with improved stroke recognition sensitivity, increased hospital prenotification, and faster tPA delivery (9). In addition to these findings, our study demonstrates that in-person interactive EMS training improves prehospital stroke identification and timely transfer to the ED. We also note that the sensitivity in our study did not change after the training, which might be explained by a relatively high baseline performance. The baseline stroke recognition sensitivity in our study (68.0%) was comparable to the post-training sensitivity in the Oostema et al. study (69.5%), suggesting a ceiling effect of stroke sensitivity improvement. Consequently, the improvement in PPV did not result in a false negative rate (type II error) increase and, thus, did not deprive stroke patients of time-sensitive treatment. Similarly, our intervention did not affect the reperfusion therapy rate. Nevertheless, our baseline IVT rate was at least two times higher (15.9%) than in 10 out of 13 studies reported in a recent meta-analysis (19). Therefore, this suggests interventions had less effect in populations with high baseline performance.

A recent attempt to enhance prehospital stroke care was undertaken in the Paramedic Acute Stroke Treatment Assessment (PASTA), a multicenter randomized clinical trial in the UK. Surprisingly, the intervention resulted in 8.5 min longer onsite care time and did not show any tPA rate improvement (17). Arguably, sophisticated prehospital assessment protocols did not facilitate IVT decision-making. On the other hand, we find conflicting results from non-randomized intervention studies showing that prehospital intervention improved reperfusion therapy rates (11, 19) and in-hospital treatment times (9, 11). In addition to the previous studies, our study shows that interactive EMS training can improve stroke recognition and prehospital transfer times and, thus, improve the overall timeliness of acute stroke care. In contrast, we did not observe changes in hospital-based metrics. However, our study was not designed to evaluate the in-hospital performance since we did not collect data on hospital prenotification rate, and the EMS staff was not involved in the clinical care after the ED admission. Other in-hospital variables, such as imaging capacity, availability of rapid image interpretation, and ED workload influence the stroke care but are not accounted for in our study.

The training effect on timely prehospital transportation was more robust in the urban compared to the suburban EMS group. Since transport time from suburban regions is longer due to greater distances between the patient and the CSC, fewer patients could arrive within 90 min of symptom onset. Furthermore, due to the national regulatory changes during the study, all suspected stroke cases were to be transported to stroke-ready hospitals, irrespective of the time of symptom onset. Thus, the number of stroke alerts outside the acute treatment window increased in the suburban but not the urban EMS group. Hence, the actual training effect in the suburban EMS group was confounded by these regulatory changes.

The recent stroke triage changes in Lithuania were aimed to increase EVT access to patients in suburban areas by transporting suspected stroke cases directly to stroke-ready centers. However, the choice between drip and ship or mothership model is context-specific (29) and poses thorny clinical dilemmas (30). EVT has a remarkable treatment effect with the number needed to treat of 2.6 to reduce disability in the early hours after stroke onset (31). If large vessel occlusion (LVO) is suspected, direct transfer to a CSC with EVT capacity might be privileged, as a shorter time to reperfusion would improve the treatment effect (32). On the other hand, bypassing primary stroke centers with IVT capacity might cause unnecessary delays to IVT and an increase in false positive large vessel occlusion transfers due to suboptimal triage. To address these questions, the RACECAT study was conceived in Catalonia, Spain, a first randomized clinical trial in the field (ClinicalTrials.gov, identifier: NCT02795962). After randomizing 1,401 patients, the preliminary study results showed no difference in ischemic stroke outcomes between drip-and-ship and mothership models in a highly coordinated stroke network (33). Similarly, in our study, we did not observe any change in IVT rate, whereas surprisingly there was a decrease in EVT rate. However, the comparison of IVT and EVT rates before and after the training should be made with caution. The post-training period coincides with the increased transfer rate of suspected suburban stroke cases with elongated LKW, resulting in a higher number of strokes arriving at the CSC beyond the EVT window. Another explanation could be a cyclical variation in EVT eligible cases. Nevertheless, since all stroke alerts were analyzed, the regulation change was not expected to confound the comparison of EMS stroke recognition. Future studies should evaluate the impact of the triage regulation change on reperfusion therapy accessibility and stroke outcomes that was out of scope of the current study.

Although the direct transfer to the CSC could be most beneficial for LVO patients, the FAST scale used in our study was not explicitly designed to detect LVO. In this context, a prospective study comparing eight prehospital scales for LVO identification showed that an adapted version of Gaze-Face-Arm-Speech-Time (G-FAST) had high LVO recognition accuracy similar or higher to other LVO scores (34). Moreover, improving the PPV of the stroke screening tools can increase the area where the mothership model provides the best stroke treatment outcome (29). More studies will be needed to explore the optimal LVO prediction methods to triage patients for different transfer pathways.

The main strength of our study is a prospective design and relatively large sample size. The blinding of EMS staff to the assessment allowed us to evaluate the training effect and avoid the apprehension bias, also known as the Hawthorne effect, when participants modify their behavior in response to their awareness of being observed (35). The main limitation of our study was the absence of a control group to fully evaluate the actual effect of the intervention. However, since there were no significant differences in demographic and clinical characteristics of suspected stroke cases before and after the training, the confounding by unmeasured factors was limited. Also, the overlap between the first month before and the last month after the training allowed us to compare similar calendar periods. Second, due to optional attendance, just over half of the EMS staff underwent the training. However, this rather introduces a bias toward the null, and we expect a stronger training effect with higher participation. Third, we found increased daily stroke rates in the post-training period, influenced by the change in the stroke triage regulations in suburban regions. However, the marginal increase in stroke prevalence during the post-training period could not fully explain the PPV improvement. We observed improved PPV with non-overlapping CI in the urban EMS and a weak trend in the suburban group favoring a consistent effect of EMS training across both groups. Fourth, our intervention did not target the dispatcher stroke recognition or hospital prenotification rate; thus, the inclusion of additional actors in the intervention might further improve the prehospital stroke care. Fifth, due to the emerging COVID-19 pandemic, we terminated our analysis before the national lockdown which significantly limited access to urgent and non-urgent healthcare (23). Had the study been continued and more cases were included in the post-training period, we could have expected a more significant effect on the in-hospital mortality. Also, we could not conclude on the long-term effects of the training beyond four months. Finally, our study was conducted in a very high cardiovascular risk population (20). These findings are generalizable to currently underrepresented populations with similar healthcare systems and EMS staffing patterns, including but not limited to Baltic states and Eastern European countries. Therefore, this study could inform prehospital clinical care and study design to improve prehospital stroke workflow using publicly available e-learning stroke education resources.

## Conclusions

Interactive EMS training improved the prehospital stroke recognition that was maintained during at least four consecutive months. Consequently, we found a measurable improvement in prehospital stroke transfer metrics and a trend toward decreased in-hospital mortality providing evidence for EMS training's positive effect on overall acute stroke care. The EMS training effect was more robust in the urban than the suburban EMS group. Thus, context-tailored training programs should be considered for EMS providers in different locations. Future studies should evaluate the long-term effects of the EMS training on prehospital stroke care, hospital-related outcomes, and aim to determine optimal retraining intervals.

## Data Availability Statement

The original contributions presented in the study are included in the article, further inquiries can be directed to the corresponding author.

## Ethics Statement

The studies involving human participants were reviewed and approved by Vilnius Regional Bioethics Committee. Written informed consent for participation was not required for this study in accordance with the national legislation and the institutional requirements.

## Author Contributions

LS, KM, DJ, AV, and RM: conception and design of the research. KM and RM: acquisition of the data. LS, KM, and RM: analysis and interpretation of the data and drafting the manuscript. LS, KM, AW, AV, KP, ES, KJ, AE, DJ, and RM: critical revision of the manuscript. All authors approved the final version to be published.

## Funding

Boehringer Ingelheim GmbH & Co KG Lithuania covered the relevant training expenses.

## Conflict of Interest

This study received funding from Boehringer Ingelheim GmbH & Co KG Lithuania. The funder was not involved in the study design, collection, analysis, interpretation of data, the writing of this article or the decision to submit for publication. The authors declare that the research was conducted in the absence of any commercial or financial relationships that could be construed as a potential conflict of interest.

## Publisher's Note

All claims expressed in this article are solely those of the authors and do not necessarily represent those of their affiliated organizations, or those of the publisher, the editors and the reviewers. Any product that may be evaluated in this article, or claim that may be made by its manufacturer, is not guaranteed or endorsed by the publisher.

## Footnotes

^1^Lithuanian Department of Statistics. (2020). Statistics Lithuania. Available at: https://www.stat.gov.lt/home [Accessed December 12, 2020].

^2^Institute of Hygiene, Ministry of Health of The Republic of Lithuania. (2019). Health and Healthcare Institutions in Lithuania. *https://hi.lt/uploads/pdf/leidiniai/Statistikos/LT_gyv_sveikata/leid2019.pdf* [Accessed June 21, 2021].

^3^Ministry of Health of The Republic of Lithuania. (2019). The Statement on Acute Stroke Work-up and Management Guideline Change. https://e-seimas.lrs.lt/portal/legalAct/lt/TAD/24591240ff0411e993cb8c8daaf8ff8a [Accessed June 21, 2021].

^4^Angels Initiative. (2020). The Advanced Stroke Life Support e-Learning. *https://www.angels-initiative.com/academy/emergency-services/advanced-stroke-life-support* [Accessed June 21, 2021].

^5^Lietuvos insulto asociacija. (2020). Informacija gydytojams. Available at: http://www.insultoasociacija.lt/index.php/profesionalams/gydytojams [Accessed June 15, 2021].
